# The lipopolysaccharide outer core transferase genes *pcgD* and *hptE* contribute differently to the virulence of *Pasteurella multocida* in ducks

**DOI:** 10.1186/s13567-021-00910-4

**Published:** 2021-03-04

**Authors:** Xinxin Zhao, Hui Shen, Sheng Liang, Dekang Zhu, Mingshu Wang, Renyong Jia, Shun Chen, Mafeng Liu, Qiao Yang, Ying Wu, Shaqiu Zhang, Juan Huang, Xumin Ou, Sai Mao, Qun Gao, Ling Zhang, Yunya Liu, Yanling Yu, Leichang Pan, Anchun Cheng

**Affiliations:** 1 Research Center of Avian Diseases, College of Veterinary Medicine, Sichuan Agricultural University, Chengdu, 611130 Sichuan China; 2 Key Laboratory of Animal Disease and Human Health of Sichuan Province, Chengdu, 611130 Sichuan China; 3 Institute of Preventive Veterinary Medicine, Sichuan Agricultural University, Chengdu, 611130 Sichuan China

**Keywords:** *Pasteurella multocida*, LPS, Outer core, *pcgD*, *hptE*, Virulence

## Abstract

Fowl cholera caused by *Pasteurella multocida* exerts a massive economic burden on the poultry industry. Lipopolysaccharide (LPS) is essential for the growth of *P. multocida* genotype L1 strains in chickens and specific truncations to the full length LPS structure can attenuate bacterial virulence. Here we further dissected the roles of the outer core transferase genes *pcgD* and *hptE* in bacterial resistance to duck serum, outer membrane permeability and virulence in ducks. Two *P. multocida* mutants, Δ*pcgD* and Δ*hptE*, were constructed, and silver staining confirmed that they all produced truncated LPS profiles. Inactivation of *pcgD* or *hptE* did not affect bacterial susceptibility to duck serum and outer membrane permeability but resulted in attenuated virulence in ducks to some extent. After high-dose inoculation, Δ*pcgD* showed remarkably reduced colonization levels in the blood and spleen but not in the lung and liver and caused decreased injuries in the spleen and liver compared with the wild-type strain. In contrast, the Δ*hptE* loads declined only in the blood, and Δ*hptE* infection caused decreased splenic lesions but also induced severe hepatic lesions. Furthermore, compared with the wild-type strain, Δ*pcgD* was significantly attenuated upon oral or intramuscular challenge, whereas Δ*hptE* exhibited reduced virulence only upon oral infection. Therefore, the *pcgD* deletion caused greater virulence attenuation in ducks, indicating the critical role of *pcgD* in *P. multocida* infection establishment and survival.

## Introduction

*Pasteurella multocida* (*P. multocida*), an encapsulated gram-negative bacterium, can cause endemic and epizootic diseases in a wide range of animal species, including fowl cholera (FC) in domestic and wild birds. Human infections by *P. multocida* also occasionally occur after cat or dog bites [1, 2]. *P. multocida* isolates are classified into five capsular types (A, B, D, E and F) based on capsular polysaccharide and 16 Heddleston serovars based on lipopolysaccharide (LPS) antigens. Capsular type A and Heddleston serovars 1 and 3 are most commonly associated with FC outbreaks [3–5]. *P. multocida* strains now can also be differentiated into 8 different LPS genotypes (L1–L8) based on distinct LPS outer core biosynthesis loci [6]. This new classification is achieved by PCR and thus easy to operate and more accurate for typing. FC manifests as acute or peracute systemic disease or chronic localized infection, leading to a high rate of morbidity and mortality in birds and posing a massive economic burden on the poultry industry worldwide. The clinical course of the disease usually ranges from a few hours to several days with a virulent strain, and death occurs suddenly in peracute cases [7]. Despite the economic importance of the bacterium, *P. multocida* is still an enigmatic pathogen, and the exact molecular mechanisms responsible for pathogenesis in pasteurellosis remain largely unknown. To date, only a few virulence factors, such as capsule and LPS [8, 9], and several regulators, including Hfq, Fis, Crp, and PhoP [10–13], have been shown to have roles in *P. multocida* virulence.

As a major cell wall component of gram-negative bacteria, LPS plays an essential role in the host-bacteria interaction and is referred to as endotoxin due to its ability to induce sepsis [14]. During infection with gram-negative bacteria, LPS is recognized by pathogen pattern recognition receptors (PRRs) in both extracellular and intracellular sites, which activates signalling pathways of innate immunity that stimulate the secretion of proinflammatory cytokines and interferons to defend against invasive bacteria [15]. However, when such responses become uncontrolled, overwhelming production of proinflammatory cytokines may lead to the development of immunopathology, manifesting as septic shock, tissue damage and even death. *P. multocida* LPS has been shown to be endotoxic. Stimulation with LPS from the B:2, A:1 and A:3 serotypes induces cell death in bovine leukocytes [16], and intravenous inoculation with LPS isolated from a serotype B:2 strain induces clinical signs of haemorrhagic septicaemia in buffalo [17].

*Pasteurella multocida* LPS lacks an O-antigen and is composed of three distinct regions; a hydrophobic lipid A domain, an inner core oligosaccharide and an outer core oligosaccharide. While the lipid A structure is still unknown, the nucleotide sequences of the biosynthesis genes required for assembly of the inner and outer core polysaccharides and the structures of those polysaccharides for all 16 Heddleston serovars have been determined by mass spectrometry compositional analysis over the last two decades [18]. The inner core is highly conserved and usually exists simultaneously with two different structures, termed glycoform A and glycoform B [19]. Transferase genes required for the assembly of the inner core are located at different sites in the *P. multocida* genome. In contrast, the outer core structures vary extraordinarily between the type strains representing different Heddleston serovars, except that Heddleston serovars 2 and 5 possess an identical outer core structure but show differences in the inner core with or without a phosphoethanolamine (PEtn) residue on heptose (Hep) II [20]. The transferase genes required for assembly of the outer core are diverse and located together in the chromosome between the two conserved genes *priA* and *fpg* [6].

**Figure 1:**
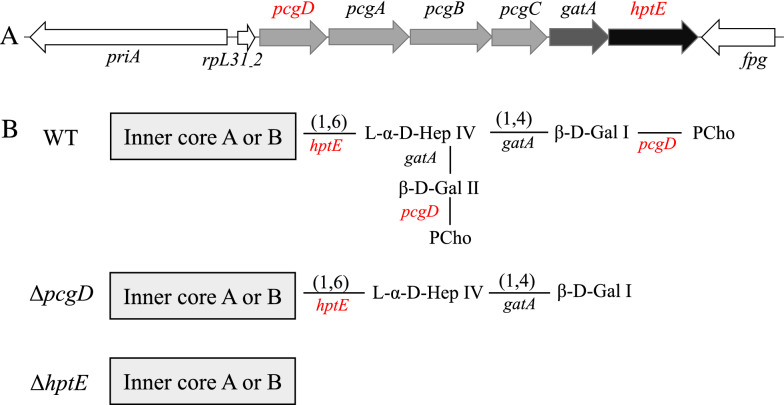
**Schematic representation of the LPS genotype L1 outer core gene locus (A) and LPS structures (B) expressed by the** ***P. multocida*** **WT and mutant strains.** The LPS structures were predicted through previous studies that uncovered the outer core structures and related synthesis genes belonging to genotype L1 of *P. multocida* [21, 22].

FC disease is frequently caused by *P. multocida* LPS genotype L1 strains. The full-length outer core of the well-studied LPS genotype L1 strain VP161 consists of Hep IV linked to galactose (Gal) I and Gal II at the 4 and 6 positions, respectively, and one phosphocholine (PCho) molecule attached to each Gal residue [21]. The synthesis of the outer core is achieved by the transferases HptE (adding Hep IV to the inner core), GatA (transferring both Gal I and Gal II to Hep IV) and PcgD (transferring PCho to both Gal residues) (Figure 1). Interestingly, inactivation of the phosphocholine pathway in VP161 also leads to the loss of a galactose from the end of the LPS structure so that the corresponding LPS lacks PCho and one galactose residue [22]. Moreover, decoration with PEtn attached to both Gal residues via the transferase PetG also exists in another LPS genotype L1 strain X73 [23]. The absence of PEtn decoration on Gal residues in VP161 is due to a single base deletion in the *petG* gene, which is located outside the outer core gene locus [23]. Previous studies have shown that the complete LPS molecule is important for LPS genotype L1 strains to display full virulence in chickens. Mutations in the genes required for decoration of LPS with PCho or PEtn, including *pcgC*, *petL* or *petK* rather than *petG*, significantly increase bacterial susceptibility to chicken antimicrobial peptide but have limited effects on bacterial virulence [22, 23]. Nevertheless, mutation of LPS biosynthesis genes for the main oligosaccharide extension of the inner core (*gctB*, *hptD* and *hptC*) or outer core (*hptE* and *gatA*) not only gives rise to reduced defence against the antimicrobial peptide but also results in bacterial growth defects in vivo and virulence attenuation to various extents [19, 21, 24]. These mutants, except Δ*hptC*, are only partially attenuated in virulence and can still induce FC symptoms in chickens. The mutants, including Δ*hptE*, Δ*gatA*, Δ*hptD* and Δ*gctB*, were shown to transform into wild-type (WT) revertants in vivo post-infection, which might eventually contribute to animal death [21]. The revertants emerged from the instability of the single-crossover insertional mutagenesis or the signature-tagged mutagenesis method used for mutant constructions [21, 24]. Thus, it is of interest and importance to further measure the virulence of more stable double crossover mutants within each of these genes. Moreover, the animal species is an important factor influencing the roles of LPS synthesis genes in the virulence of *P. multocida* [25]. Therefore, it is also necessary to evaluate the function of *P. multocida* LPS in other avian species.

In this study, two LPS outer core transferase genes, *pcgD* and *hptE*, were deleted in the WT *P. multocida* strain PM0818 via double-crossover homologous recombination, and then the abilities of the mutants were investigated and compared with the WT strain in response to duck serum complement and a variety of hydrophobic antibiotics in vitro; to colonize tissues, and to cause pathological lesions and death in ducks.

## Materials and methods

### Bacterial strains and growth conditions

The bacterial strains and plasmids used are shown in Table 1. All the mutant strains were derived from the virulent WT *P. multocida* A:1 strain PM0818 with a muscular 50% lethal dose (LD_50_) of < 100 CFU and an oral LD_50_ of approximately 10^6^ CFU in ducklings [10, 13]. *P. multocida* strains were grown at 37 °C in brain heart infusion (BHI) broth or on BHI agar (BD Bioscience, USA), and *Escherichia coli* (*E. coli*) strains were grown in Luria–Bertani (LB) broth or on LB agar. Tryptic soybean agar (TSA, Difco Laboratories, USA) was generally used for colony counts of *P. multocida* strains. When required, antibiotics were added to the medium at the following concentrations: kanamycin, 50 μg/mL; chloramphenicol, 25 μg/mL.

**Table 1:** **Bacterial strains and plasmids used in this study**

Strains or plasmids	Description	Source
Plasmids		
pMC-Express	A broad host-range shuttle vector derived from pMIDG100, *sodC* promoter, Cm^r^	[27]
pRE112	*sacB* mobRP4 R6K ori Cm^r^	[26]
pCZ51	pRE112-Δ*pcgD*	This work
pCZ53	pRE112-Δ*hptE*	This work
pCZ54	Insertion of complete *pcgD* into pMC-Express	This work
pCZ56	Insertion of complete *hptE* into pMC-Express	This work
Strains		
SM10 *λ pir*	*E. coli thi thr*-1 *leu*6 *pro*A2 *his*-4 *arg* E2 *lac*Y1 *galK*2*, ara*14*xy*l5 *supE*44*, λ pir*	[43]
ATCC25922	*E. coli*, quality control strain for antibiotic sensitivity test	ATCC
PM0818	*P. multocida* 0818, Wild-type and virulent, LPS genotype L1	[10]
PMZ1	PM0818 Δ*pcgD*::*kanR*	This work
PMZ3	PM0818 Δ*hptE*::*kanR*	This work

### Plasmid and mutant strain construction

**Figure 2:**
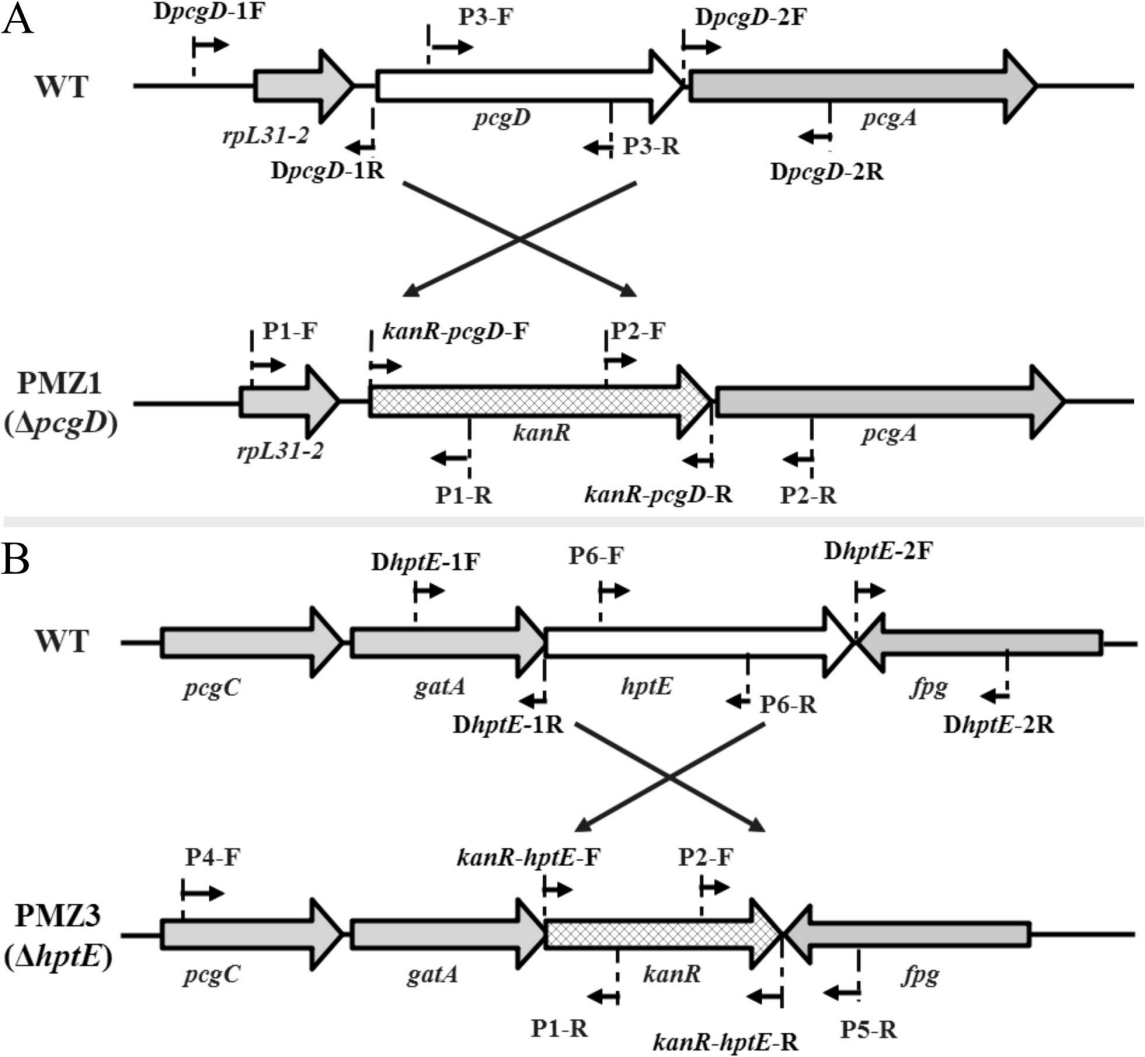
**Schematic strategy used for deletion of the target *pcgD* (A) or *hptE*** **(B) gene in *P. multocida***. The target gene was replaced with the *kanR* gene via homologous recombination. The primer pairs D*pcgD*-1F/1R, D*pcgD*-2F/2R, and *kanR*-*pcgD*-F/R were designed for the construction of PMZ1 (Δ*pcgD*) and D*hptE*-1F/1R, D*hptE*-2F/2R, and *kanR*-*hptE*-F/R for the construction of PMZ3 (Δ*hptE*). The primer pairs P1-F/R, P2-F/R, and P3-F/R were designed for the characterization of PMZ1 (Δ*pcgD*) and P4-F/P1-R, P2-F/P5-R, and P6-F/R for the characterization of PMZ3 (Δ*hptE*).

The primers used in this study are listed in Additional file 1. The *P. multocida* mutant strains were constructed by allelic exchange using the suicide T-vector pRE112 [26] as previously described [13]. The primers used for the mutant construction and characterization are depicted in Figure 2. To delete the *pcgD* gene, the primer pairs D*pcgD-*1F/1R, D*pcgD-*2F/2R and *kanR*-*pcgD*-F/R were used to amplify the upstream segment (440 bp) and downstream segment (335 bp) of the *pcgD* gene from the PM0818 genome and the kanamycin resistance (*kanR*) gene (837 bp) from the pET28a plasmid, respectively. The three gene fragments were then linked together by overlap PCR using primers D*pcgD-*1F and D*pcgD-*2R. The resulting PCR product was digested with *Kpn*I-HF and *Xma*I and ligated into pRE112 to generate the plasmid pCZ51, which carries a deletion of the entire *pcgD* gene sequence. This plasmid was subsequently introduced into *P. multocida* PM0818 from *E. coli* SM10 λ pir via conjugation, and the Δ*pcgD* mutant strain designated PMZ1 was selected on BHI agar containing kanamycin. The *pcgD* mutation was confirmed by PCR with three primer pairs (P1-F/R, P2-F/R and P3-F/R) for the deleted region and flanking DNA, and the PCR products from P1-F/R and P2-F/R were subjected to DNA sequencing (BGI-Shenzhen, China) (data not shown). The same method was applied to construct the mutant strain PMZ3 (Δ*hptE*).

To complement each gene mutation in the *P. multocida* mutants, the region (12 bp) immediately upstream of the start codon and the complete coding sequences of *pcgD* and *hptE* were amplified from the PM0818 genome with primer pairs C*pcgD*-F/R and C*hptE*-F/R, respectively. The resulting *pcgD* or *hptE* segment was inserted into the *Kpn*I and *Not*I sites of the shuttle plasmid pMC-Express [27] to generate the complementation plasmid pCZ54 or pCZ56. Then, the recombinant plasmids were transformed into the corresponding mutant strains, generating two complemented strains, PMZ1 (pCZ54) and PMZ3 (pCZ56). Moreover, the empty plasmid pMC-Express was also transformed into the mutant strains, generating the control strain, PMZ1 (pMC-Express) or PMZ3 (pMC-Express).

### Phenotype detection

To determine the relative growth rate of each *P. multocida* strain grown under normal in vitro growth conditions, overnight bacterial cultures were diluted to an optical density (OD) = 0.05 in fresh BHI medium, and then the bacterial growth was determined by measuring the OD_600_ value every 0.5 h during the first 0 h-2 h and every 2 h from 3 to 15 h at 37 °C. To detect the LPS phenotype, whole-cell lysates of the *P. multocida* WT strain or mutants were subjected to sodium dodecyl sulphate–polyacrylamide gel electrophoresis (SDS-PAGE) on 15% (w/v) acrylamide gels using a Tricine-SDS buffer system (Bio-Rad Laboratories, California, USA) followed by silver staining as previously described [28].

### Susceptibility to duck serum

The bactericidal activity of duck serum complement against the *P. multocida* strains was measured as previously described [10]. In brief, the *P. multocida* WT and mutant strains were grown to the mid-log growth phase (OD = 0.6) in BHI broth and re-suspended in phosphate-buffered saline (PBS) at a concentration of 10^4^ CFU/mL. The bacterial suspensions were then treated with 90% normal duck serum or heat-inactivated duck serum for 3 h at 37 °C with shaking. After incubation, serial dilutions of the samples in PBS were cultured on TSA plates at 37 °C overnight. The survival rate of each strain was calculated as the CFU of normal serum group divided by the CFU of the inactivated serum group.

### NPN assay

Outer membrane (OM) permeability was determined by utilizing N-phenyl-1-naphthylamine (NPN) (Sigma-Aldrich, USA), which is a hydrophobic fluorescent probe, as described previously, with minor modification [29]. In brief, the *P. multocida* WT and mutant strains were grown to the mid-log phase and then harvested by centrifugation and resuspended in PBS to an OD_600_ of 0.5. The NPN dissolved in acetone was added to the suspension at a final concentration of 80 µM. Control samples without added cells were also treated with NPN. Then, the fluorescence of the mixture was measured immediately using a spectrofluorometer (Thermo Scientific Varioskan Flash). The excitation wavelength, emission wavelength and slit width used were 350 nm, 420 nm, and 5 nm, respectively.

### The minimum inhibitory concentrations (MICs) of antibiotics

The MICs of several hydrophobic antibiotics (Dalian Meilun Biotechnology, China) and SDS (Sigma-Aldrich), including azithromycin, novobiocin, spiramycin, ciprofloxacin, rifampicin, and nalidixic acid, against the *P. multocida* WT and mutant strains were measured using the standard microscale broth dilution method performed according to the Clinical and Laboratory Standard Institute criteria as previously described [30]. The *E. coli* ATCC25922 strain was included as quality control.

### Colonization and virulence of *P. multocida* strains and resultant histopathology changes in ducks

One-day-old partridge Sichuan ducklings were purchased from Grimaud Breeding Co., Ltd. (Chengdu, China) and acclimated for several days after arrival. To measure the colonization of the mutants, 7-day-old ducks were orally inoculated with approximately 10^9^ CFU of the *P. multocida* WT strain or each mutant strain (6 ducks/group). Then, blood and tissues, including the lung, spleen and liver, were collected from animals 12 h and 24 h after infection. Each tissue sample was weighed and homogenized in 1 mL of PBS. Next, the suspension or the collected blood was serially diluted, and the appropriate dilution was plated onto TSA to determine the number of viable bacteria. The colonization level is represented as CFU per gram of tissue (CFU/g) or CFU per mL of blood (CFU/mL).

Moreover, to observe histopathological changes, the spleens and livers randomly collected from 4 ducks within each group in the colonization analysis at 24 h were fixed in 4% paraformaldehyde, dehydrated, embedded in paraffin, sectioned into 4-μm-thick sections, placed on microscope slides, and stained with haematoxylin and eosin (HE) using standard procedures. Four ducks inoculated with the PBS were also included as a negative control. Scoring with light microscopy evaluation was based on the severity of pathological changes in terms of tissue structure, congestion, inflammatory infiltration, haemorrhage, degeneration and necrosis. Scores ranging from 0 to 4 represent different levels of significant pathological lesions and were assessed by three diagnostic pathologists. Furthermore, to determine bacterial virulence, 7-day-old ducks were challenged with approximately 10^9^ CFU of each *P. multocida* strain orally or 100 CFU intramuscularly (10 ducks/group). Then, the survival of ducks in each infected group was observed and recorded in a 7-day period.

### Statistical analysis

The data are shown as the means ± SDs and were analysed by one-way ANOVA followed by Dunnett’s multiple comparison test in GraphPad Prism (GraphPad Software, California, USA). The survival curves in the virulence assay were analysed by the Log-rank test in GraphPad Prism. A probability value of *p* < 0.05 was considered statistically significant. The animal experiments were performed twice, and the in vitro experiments were conducted in triplicate independently at least three times.

## Results

### Construction and characterization of *P. multocida* mutants

The LPS genotype L1 outer core biosynthesis locus of *P. multocida* contains seven genes, *rpL31_2*, *pcgD*, *pcgA*, *pcgB*, *pcgC*, *gatA* and *hptE*, among which *rpL31_2* is unrelated to LPS biosynthesis (Figure 1A). Genome sequencing and bioinformatics analyses showed that the outer core locus of the WT strain PM0818 (GenBank accession number MT542700) was intact and highly conserved compared with the well-studied LPS genotype L1 strains VP161 and X73. Additionally, a homologue of *petG* (responsible for the addition of PEtn to the Gal residues) was present in the PM0818 genome but was a pseudogene because of a one-base deletion at nucleotide 1076 compared with the functional *petG* gene of the X73 strain (Additional file 2), similar to previously reported observations for the nonfunctional *petG* gene of the VP161 strain [23]. Thus, we predicted that PM0818 possessed an outer core structure identical to the VP161 strain, without PEtn attached to the Gal residues (Figure 1B).

**Figure 3:**
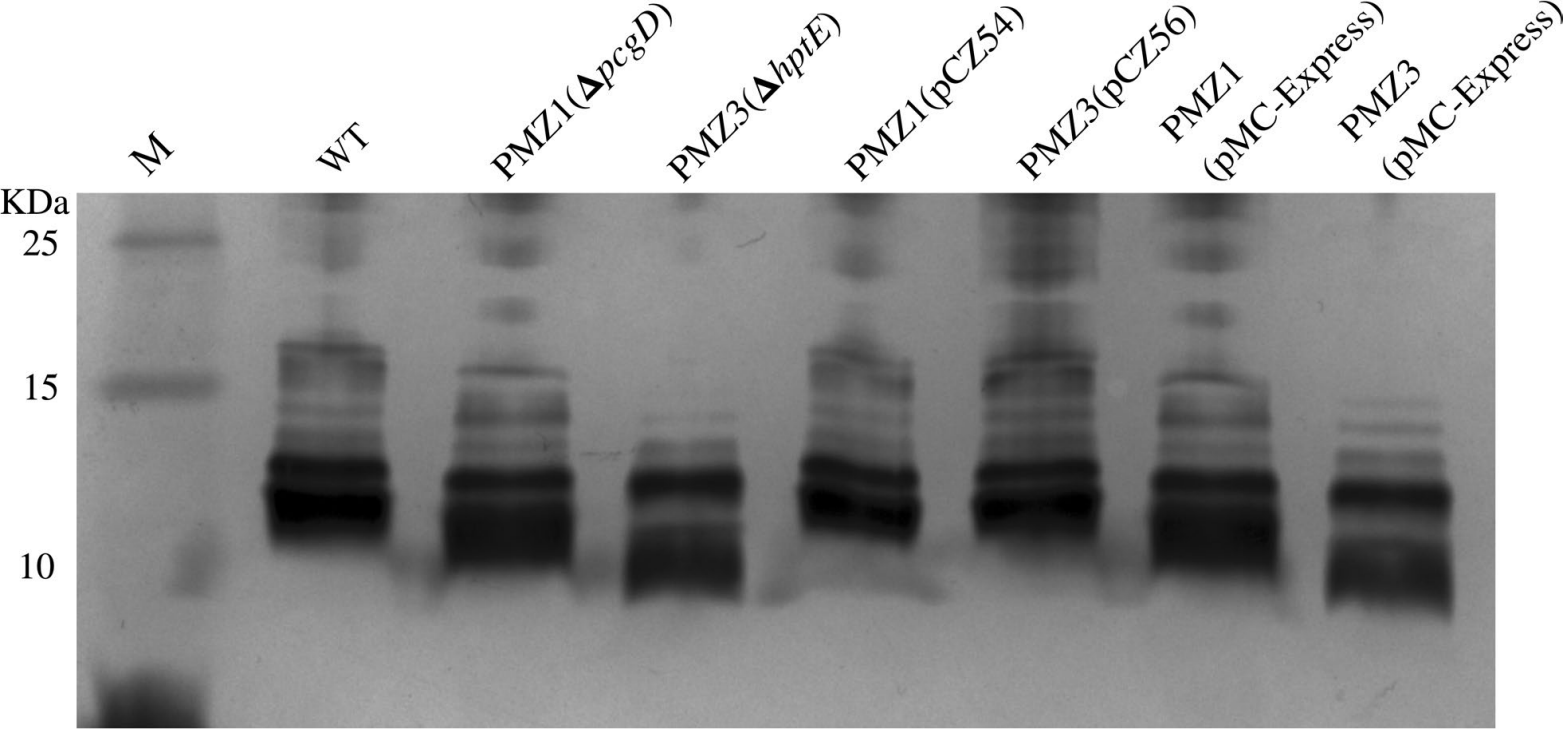
**LPS phenotypes.** LPS extracted from the *P. multocida* WT strain, two mutant strains, PMZ1 (Δ*pcgD*) and PMZ3 (Δ*hptE*), and two complemented strains, PMZ1 (pCZ54) and PMZ3 (pCZ56), and two control strains, PMZ1 (pMC-Express) and PMZ3 (pMC-Express), were subjected to SDS-PAGE followed by silver staining. M refers to the protein marker.

The outer core transferase genes, *pcgD* and *hptE*, were deleted in WT strain PM0818 by suicide plasmid-based homologous recombination, as depicted in Figures 2A and B, generating two mutants termed PMZ1 (Δ*pcgD*) and PMZ3 (Δ*hptE*), respectively. The genotypes of the mutants were verified via PCR using several pairs of primers. The DNA segment containing the upstream sequence of *pcgD* or *hptE* and a partial *kanR* cassette (P1-F/R or P4-F/P1-R) and the segment containing the downstream sequence of *pcgD* or *hptE* and a partial *kanR* cassette (P2-F/R or P2-F/P5-R) were present in the mutant strain but not in the WT strain, whereas the partial sequence of *pcgD* or *hptE* (P3-F/R or P6-F/R) was only amplified from the WT strain (Additional files 3A and 3B). Next, two complementation plasmids, pCZ54 encoding *pcgD* and pCZ56 encoding *hptE*, were constructed and transformed into the corresponding mutant strain, generating two complemented strains, PMZ1 (pCZ54) and PMZ3 (pCZ56). Moreover, each mutant harbouring the empty plasmid pMC-Express was also constructed as a control. The outer core structures of the two mutants were predicted according to previous studies whereby mass spectrometry and nuclear magnetic resonance were performed to determine exact LPS core structures (Figure 1B) [21]. To confirm the prediction, LPS phenotypes of the *P. multocida* strains were detected by silver staining. As expected, genetic inactivation of *pcgD* or *hptE* led to the production of truncated LPS, as demonstrated by the observation that the LPS from the two mutants migrated further within the gel than WT LPS. The LPS of PMZ3 (Δ*hptE*), with the shorter oligosaccharide extension, moved further than that of Δ*pcgD* (Figure 3). Moreover, the mutants carrying the empty plasmid also produced truncated LPS phenotypes, while complementation of each mutation with the appropriate gene provided in *trans* resulted in the production of a longer LPS molecule compared with the LPS produced by the corresponding mutant (Figure 3), suggesting the production of functional PcgD or HptE protein and formation of the full-length LPS outer core in the complemented strain. Additionally, the growth curves of the mutants in vitro were similar to those of the WT strain at 37 ℃ (Additional file 4).

### Susceptibility to duck serum and outer membrane permeability of the *P. multocida* mutants

Loss of the full-length LPS outer or inner core in *P. multocida* was previously shown to increase susceptibility to the antimicrobial peptide fowlicidin-1 rather than chicken serum complement [21]. Here we evaluated the role of the LPS outer core genes *pcgD* and *hptE* in bacterial resistance to duck serum complement and OM permeability using the probe NPN. The mutants PMZ1 (Δ*pcgD*) and PMZ3 (Δ*hptE*) showed similar resistances to duck serum complement (Additional file 5) and comparable NPN fluorescence signals to the WT strain (Additional file 6). Additionally, compared with the WT strain, the two mutants had similar susceptibilities to SDS and a subset of hydrophobic antibiotics, including azithromycin, novobiocin, spiramycin, ciprofloxacin, rifampicin, nalidixic acid and bacitracin (Additional file 7). These results indicated that the bacterial resistance to duck serum and OM integrity was not affected by the truncation of the LPS structure due to the inactivation of the outer core transferase genes *pcgD* or *hptE*.

### Colonization of *P. multocida* mutants and resultant pathological lesions in ducks

**Figure 4:**
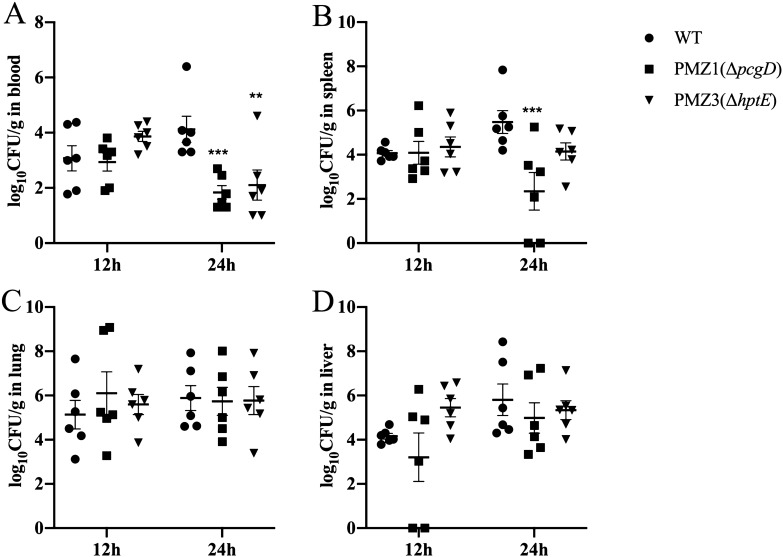
**Colonization in ducks.** Groups of ducklings (*n* = 6/group) were inoculated orally with approximately 10^9^ CFU of the *P. multocida* WT strain or each mutant strain. The blood (**A**), spleen (**B**), lung (**C**) and liver (**D**) were collected at 12 h or 24 h post-infection, and the bacterial loads of each strain were calculated as CFU/mL in the blood and CFU/g in the three tissues. The asterisk above the error bar indicates significance compared with the WT group. **, *p* < 0.01; ***, *p* < 0.001.

To detect the effects of *pcgD* or *hptE* mutation on bacterial colonization, approximately 10^9^ CFU of each mutant strain or the WT strain was inoculated orally into seven-day-old ducks, and the bacterial loads in the blood, spleen, liver and lung were measured 12 h and 24 h post-infection. Compared with the WT strain, the colonization levels of the two mutants were not changed in the blood or all detected tissues at 12 h post-infection (Figures 4A–D); however, at 24 h post-infection, both mutants colonized the blood at a significantly lower level than the WT strain (Figure 4A). Additionally, the bacterial counts of PMZ1 (Δ*pcgD*) decreased significantly in the spleen rather than the liver and lung, while the counts of PMZ3 (Δ*hptE*) were similar to those of the WT strain in all three tissues (Figures 4B–D). This result indicated that truncation of the LPS due to deletion of the *pcgD* or *hptE* gene had a significantly negative effect on the colonization ability of *P. multocida* in the blood, and the *pcgD* mutation further attenuated bacterial colonization in the spleen of ducks.

**Figure 5:**
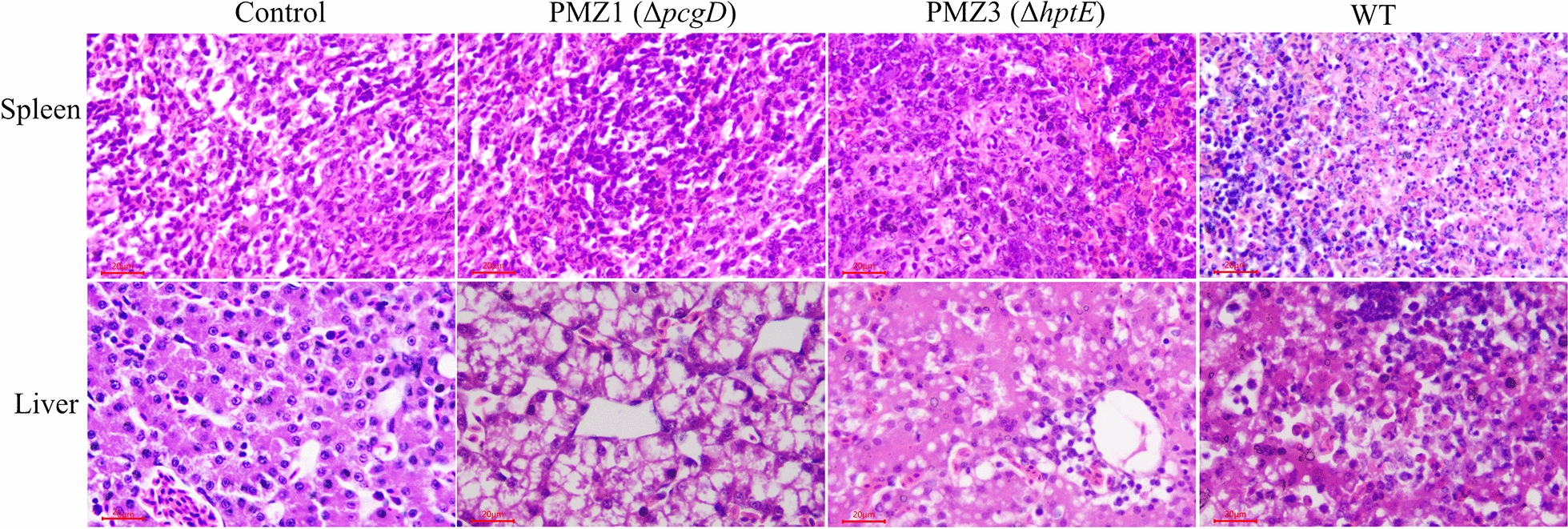
**Pathological changes in ducks.** Groups of ducklings (*n* = 4/group) were infected orally with approximately 10^9^ CFU of the *P. multocida* WT strain or each mutant strain or with control PBS. The histopathological lesions in the spleen and liver caused by each bacterial strain were analysed by HE staining.

Pathological changes in the spleen and liver were also detected by HE staining at 24 h after infection. The ducks in the PBS control group showed normal tissue structures without histopathological lesions, while the WT strain-infected ducks displayed severe focal necrosis with abundant fragmented nuclei remaining and infiltration of a number of heterophilic granulocytes and structural disorder in the spleen and the liver (Figure 5). The *pcgD* or *hptE* mutation alleviated infection-induced tissue lesions to some extent. Neither PMZ1 (Δ*pcgD*) nor PMZ3 (Δ*hptE*) infection induced necrosis and merely resulted in significant granulocyte infiltration and slight congestion in the spleen; the former induced only hepatocyte degeneration, while the latter induced moderate granulocyte infiltration and inflammatory exudation in the liver (Figure 5). Of note, the changes in histopathological scores were highly coincident; deletion of *pcgD* or *hptE* significantly reduced splenic lesions, and inactivation of *pcgD* but not *hptE* weakened the pathological liver lesions (Table 2).

**Table 2:** **Histopathological scores in different infection groups**

Groups^c^	WT	PMZ1 (Δ*pcgD*)	PMZ3 (Δ*hptE*)
Spleen	3.5 ± 0.3^a^	2.0 ± 0.4^b^	2.0 ± 0.4^b^
Liver	3.8 ± 0.5^a^	2.3 ± 0.3^b^	3.5 ± 0.3^a^

^a, b^The different letters in each row represent significant differences between indicated groups.

^c^The scores in the table were assessed from 4 ducks per infection group and represented as the means ± SDs.

### Virulence of the *P. multocida* mutants in ducks

**Figure 6:**
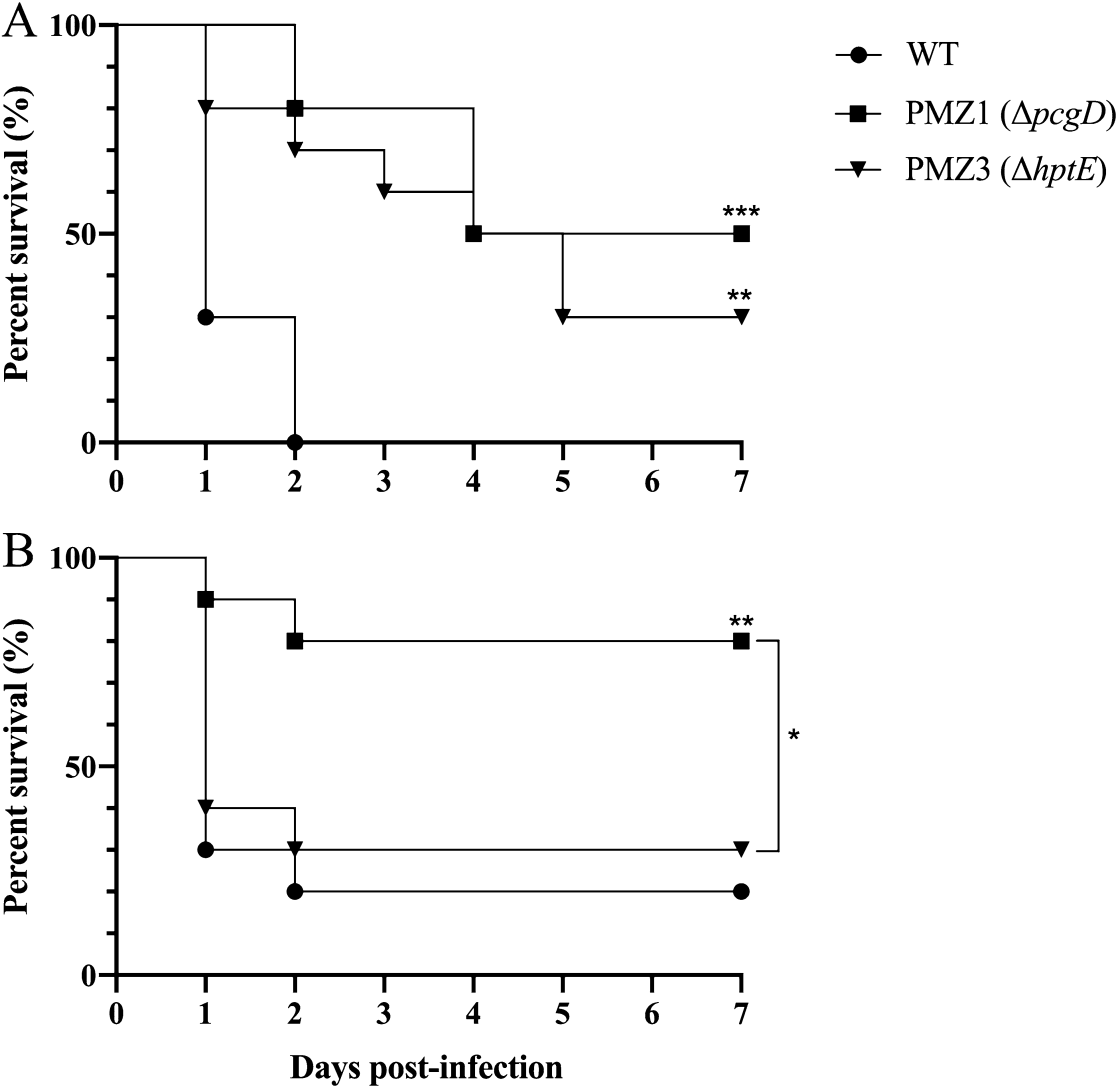
**Virulence trial.** Groups of ducklings (*n* = 10/group) were challenged with approximately 10^9^ CFU of the *P. multocida* WT strain or each mutant strain orally (**A**) or with 100 CFU of each strain intramuscularly (**B**). Then, the animal survival in each challenge group was monitored. *, *p* < 0.05; **, *p* < 0.01; ***, *p* < 0.001.

The virulence of the *P. multocida* strains was determined by inoculation of ducks with approximately 10^9^ CFU of each bacterial strain orally or 100 CFU intramuscularly. The ducks infected with the WT strain showed 100% death following oral inoculation and 20% survival following intramuscular inoculation, whereas the PMZ1 (Δ*pcgD*) and PMZ3 (Δ*hptE*) challenges resulted in 50% and 30% survival following oral inoculation (Figure 6A) and 80% and 30% survival following intramuscular inoculation, respectively (Figure 6B). The log-rank test showed that compared with the WT strain, PMZ1 (Δ*pcgD*) was significantly attenuated in virulence by both the oral and intramuscular routes, while the virulence of PMZ3 (Δ*hptE*) was reduced only in the oral infection route. Moreover, PMZ1 (Δ*pcgD*) was less virulent than PMZ3 (Δ*hptE*) during intramuscular infection (Figure 6B).

## Discussion

FC is one of the most important diseases, leading to massive economic losses to the poultry industry worldwide; however, the underlying mechanisms of the high mortality and severe tissue damage caused by the causative agent, *P. multocida*, are still poorly understood [7]. Revealing such mechanisms is essential for developing effective live attenuated vaccines against pasteurellosis, such as the *P. multocida* auxotrophic *aroA* mutant and the *aroA* mutant carrying a deletion of a certain LPS synthesis gene [31, 32]. The polysaccharide components of LPS have been shown to be closely associated with the pathogenicity of *P. multocida* in chickens [18]; nevertheless, their roles in virulence have not been studied in other avian species. Here we constructed two defined *P. multocida* mutants with deletions of the outer core glycotransferase genes, *pcgD* or *hptE*, via double-crossover homologous recombination, and we evaluated their resistance to duck serum, outer membrane permeability, and their colonization and virulence in ducks, another natural host. The *gatA* is another transferase gene involved in the synthesis of the LPS outer core and must be studied, unfortunately, however, we did not successfully construct the *gatA* mutant strain in the WT strain.

It was found that the LPS outer core was irrelevant for the stress response to duck serum, which is in agreement with previous studies showing that *P. multocida* mutants with incomplete LPS inner cores or without PEtn on lipid A show the same level of serum resistance as the WT parent strain [23, 24]. It also further supports a previous finding showing that the resistance of *P. multocida* to serum complement is dominantly mediated by the capsule rather than by LPS [33]. The OM of gram-negative bacteria is composed of the outer LPS layer and a phospholipid inner leaflet that confers a semipermeable barrier function against toxic molecules [34]. The loss of lipid A synthesis or destruction of the LPS core region or porin proteins has been previously shown to increase membrane permeability and a range of antibiotics in several gram-negative bacteria [35–38]. Our results demonstrated that the two outer core mutants maintained normal OM permeability. The irrelevance of the LPS outer core and OM integrity might be a consequence of the outer core being the most distal and exposed region of the LPS molecule. It would be of interest to investigate whether the LPS inner core or lipid A is closely associated with the OM permeability of *P. multocida*.

The capacities of the two *P. multocida* mutants to colonize tissues and induce histopathological changes and animal mortality were assessed in ducks. Both strains showed defective growth in the blood, decreased pathological splenic lesion induction and increased animal survival in comparison to those of the WT strain after high-dose oral infection. Nevertheless and interestingly, the virulence attenuation exhibited by PMZ1 (Δ*pcgD*) was greater than that exhibited by PMZ3 (Δ*hptE*), as demonstrated by the finding that deletion of *pcgD* but not *hptE* led to reduced bacterial colonization in the spleen and alleviated liver injury, and attenuated virulence upon intramuscular infection. The defective in vivo growth of the Δ*pcgD* and Δ*hptE* was similar to previous virulence studies on *P. multocida* in chickens to some extent [21, 22]; however, the virulence attenuation by intramuscular injection exhibited by the Δ*pcgD* was somewhat contradictory to a previous study in which all the chickens injected with 60 CFU of a PCho mutant (*pcgC* mutant) showed symptoms of FC but did not succumb to disease, indicating that the addition of PCho to A:1 LPS was not essential for the virulence of *P. multocida* in chickens [22]. As the four genes of the *pcgDCBA* locus work together for assembly of PCho on the outer core [18], the discrepancy triggered by the two mutants might be due to differences in the inherent nature of chickens and ducks, or the possibility that *pcgD* has functions beyond PCho synthesis.

The virulence assay with the *hptE* mutant in chickens resulted in a delayed onset of fowl cholera symptoms in the presence of wild-type revertants post-intramuscular infection [21]. Our study further confirmed the comparable virulence of the stable Δ*hptE* with the WT strain upon intramuscular challenge. Inactivation of *hptE* results in the loss of the full outer core structure and was also found to produce a novel outer core extension comprising β-Gal-(1–4)-β-GlcNAc-(1–3)-β-Gal-(1–3)-β-GlcNAc-(1–4) that is present in approximately 15% of the LPS molecules [21], which might compensate for the loss of the original outer core and contribute to high virulence in ducks. LPS is a strong stimulus for the production of innate immunity by binding to PRRs, while large amounts of LPS can also cause excessive proinflammatory responses that can lead to sepsis and even death [15]. Although lipid A is the main ligand for TLR4/MD-2, parts of the LPS polysaccharide backbone are also involved in the binding of the TLR4 ectodomain [39, 40], and the core oligosaccharide of the *Pseudomonas aeruginosa* LPS has been proven to be a kind of pathogen-associated molecular pattern recognized by the innate immune system, leading to host bactericidal defences [41]. The different LPS structures produced by Δ*pcgD* and Δ*hptE* might stimulate dissimilar inflammatory responses that partially led to the distinct consequences in virulence phenotypes displayed by the two mutants. To determine whether the innate immunity was altered, the cytokine profiles induced by purified LPS from the WT strain, the *pcgD* mutant, and the *hptE* mutant need to be evaluated both in vitro and in vivo. Furthermore, the colonization of both mutant strains was markedly decreased in the blood from 12 to 24 h post-infection, suggesting a profound clearance of the bacteria by the innate immunity in this period. As the *pcgD* or *hptE* mutation did not affect bacterial tolerance to the duck serum, it is worth detecting the susceptibility of both mutants to phagocyte ingestion and intracellular killing in the future, which are major components of innate immunity [42].

In conclusion, our study systematically investigated the role of two outer core transferase genes, *pcgD* and *hptE*, in susceptibility to duck serum, OM permeability and the virulence of *P. multocida* in ducks. The two gene mutations had no adverse effect on bacterial resistance to duck serum complement and OM permeability. Mutations in *pcgD* or *hptE* reduced bacterial virulence to a different extent compared with the WT strain. PMZ3 (Δ*hptE*) showed compromised colonization in only the blood, moderate splenic lesions, and severe liver injuries as well as reduced virulence via the oral route rather than the intramuscular route, whereas PMZ1 (Δ*pcgD*) displayed decreased colonization in the blood and spleen, moderate splenic and liver lesions and further reduced virulence via both the oral and intramuscular route. Therefore, deletion of the *pcgD* gene led to greater *P. multocida* virulence reduction in ducks.

## Supplementary Information

**Additional file 1. Primers used in this study.**

**Additional file 2. Alignments of the gene sequences of *petG*** **among three genotype L1 strains, PM0818, VP161 (NZ_CP048792, PmVP161_RS04065) and X73 (NZ_CM001580, X73_RS06225).** PM0818 *petG* shared > 99% identity with the homologs of the VP161 and X73 but was also a pesudogene due to a single base deletion at nucleotide 1076 like the VP161 *petG* compared with the homolog of the X73 strain. The blue and red indicate the positions of the point mutation and the base deletion, respectively.

**Additional file 3. Characterization of the *P. multocida*** **mutant strains via PCR.** The WT strain and PMZ1 (Δ*pcgD*) mutant were identified using primers P1-F/R, P2-F/R, and P3-F/R to confirm the *pcgD* mutation; similarly, the WT and PMZ3 (Δ*hptE*) were identified using primers P4-F/P1-R, P2-F/P5-R, and P6-F/R to confirm the *hptE* mutation. M refers to the DNA marker.

**Additional file 4. The growth curves.** The *P. multocida* strains were grown in BHI medium overnight, and the cultures were diluted to an OD600 of 0.05 in fresh medium. Then the bacterial growth was determined by measuring the OD600 value every 0.5 h during the first 0 h-2 h and every 2 h from 3 to 15 h at 37 °C.

**Additional file 5. Serum bactericidal assay.** The* P. multocida* strains were cultured in media to an OD600 of 0.6 and diluted to a final concentration of 104 CFU/mL in PBS. Then, the bacteria were mixed with 90% normal duck serum or heat-inactivated duck serum and incubated for 3 h at 37 °C. After incubation, serial dilutions of the samples were spread on TSA plates for counting. The survival rate of each strain was calculated as the CFU after active serum treatment divided by the CFU after the heat-inactivated serum treatment.

**Additional file 6. NPN assay.** The log phase growth medium of the *P. multocida* strains was harvested, and the bacteria were resuspended in PBS at an OD600 of 0.5 with 80 µM NPN. PBS without bacteria was used as a control. The fluorescence of the suspension was measured immediately using a spectrofluorometer.

**Additional file 7. The MICs of SDS and hydrophobic antibiotics.**

## Data Availability

All datasets are presented in the paper or additional files supporting the manuscript.

## Abbreviations

*P. multocida*: *Pasteurella multocida*
FC: Fowl cholera
LPS: Lipopolysaccharide
PRR: Pathogen pattern recognition receptor
PEtn: Phosphoethanolamine
Hep: Heptose
Gal: Galactose
PCho: Phosphocholine
WT: Wild-type
BHI: Brain heart infusion
*E. coli*: *Escherichia coli*
LB: Luria–Bertani
TSA: Tryptic soybean agar
OD: Optical density
PBS: Phosphate buffered saline
Cm: Chloramphenicol
Amp: Ampicillin
Kan: Kanamycin
CFU: Colony forming unit
SDS-PAGE: Sodium dodecyl sulphate–polyacrylamide gel electrophoresis
OM: Outer membrane
NPN: N-phenyl-1-naphthylamine
MIC: Minimum inhibitory concentration
