# Supplementary material for: The lipopolysaccharide outer core transferase genes pcgD and hptE contribute differently to the virulence of Pasteurella multocida in ducks
Source: Vet Res. 2021 Mar 4;52:37. doi: 10.1186/s13567-021-00910-4 (PMC7931556; doi:10.1186/s13567-021-00910-4)
Supplement: Supplementary file 1 — Additional file 1. Primers used in this study. [file 13567_2021_910_MOESM1_ESM.docx]

**Additional file 1 Primers used in this study**

| Primer | Sequence 5’-3’ |
| --- | --- |
| D*pcgD*-1F | GCCACCTTACATACAAACTG |
| D*pcgD*-1R | GAATATGGCTCATGAAGTCTCCTTACATGTA |
| D*pcgD*-2F | AGTTTTTCTAACATACTTATGAATATACAAAATT |
| D*pcgD*-2R | GTACTATGATCTAGGCTAAAACTG |
| D*hptE*-1F | CTGGAAGATGATGCTATCG |
| D*hptE*-1R | ATGGCTCATGCTTTTTATCATTTCAAACCCGCTC |
| D*hptE*-2F | ATCTTATCTCTTCTGACACTTGGGGC |
| D*hptE*-2R | TCGCCGTGCGAAGTATTTAA |
| *kanR*-*pcgD*-F | TAAGGAGACTTCATGAGCCATATTCAACGGGA |
| *kanR*-*pcgD*-R | CATAAGTATGTTAGAAAAACTCATCGAGC |
| *kanR*-*hptE*-F | GATAAAAAGCATGAGCCATATTCAACGGGAAACG |
| *kanR*-*hptE*-R | GTGTCAGAAGAGATAAGATTAGAAAAACTCATCGAGC |
| C*hptE*-F | GGGGTACCAGCGGGTTTGAAATGATA |
| C*hptE*-R | TTGCGGCCGCCTATTTGACAAGCCATGC |
| C*pcgD*-F | GGGGTACCGTAAGGAGACTTATGAAGT |
| C*pcgD*-R | TTGCGGCCGCTTATTTACGACGGAATTC |
| P1-F | TGGAAGTTACGGCAACTTGTTCTTGC |
| P1-R | TGCATCATCAGGAGTACG |
| P2-F | CGTAATGGCTGGCCTGTTGAACAAG |
| P2-R | TATCTCCCTCTACTAGGAAG |
| P3-F | CATAAGTCTGCTCCTCTTGC |
| P3-R | GTGATCGGGTGTATTATCTGG |
| P4-F | TGTAACAGGACATCTGTCAG |
| P5-R | GTGCTGTCTAACGCAATCC |
| P6-F | GAAGCATCCTCTTCCTCCGC |
| P6-R | ACTTCTGGGTTGCCAATGTG |
