# Supplementary figures and images for: The lipopolysaccharide outer core transferase genes pcgD and hptE contribute differently to the virulence of Pasteurella multocida in ducks
Source: Vet Res. 2021 Mar 4;52:37. doi: 10.1186/s13567-021-00910-4 (PMC7931556; doi:10.1186/s13567-021-00910-4)

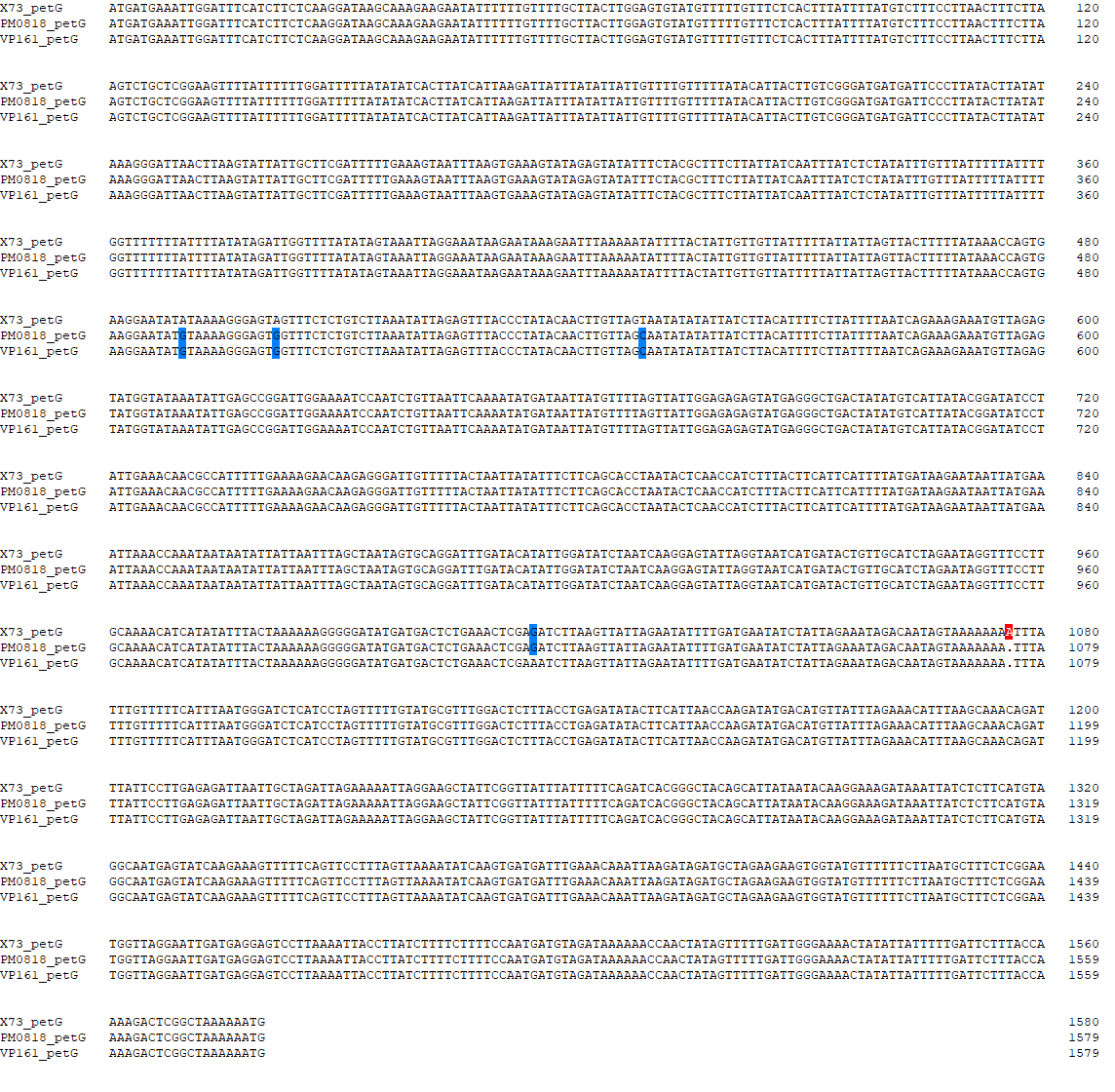

Supplement: Supplementary file 2 — Additional file 2. Alignments of the gene sequences of petG among three genotype L1 strains, PM0818, VP161 (NZ_CP048792, PmVP161_RS04065) and X73 (NZ_CM001580, X73_RS06225). PM0818 petG shared > 99% identity with the homologs of the VP161 and X73 but was also a pesudogene due to a single base deletion at nucleotide 1076 like the VP161 petG compared with the homolog of the X73 strain. The blue and red indicate the positions of the point mutation and the base deletion, respectively. [file 13567_2021_910_MOESM2_ESM.tif]

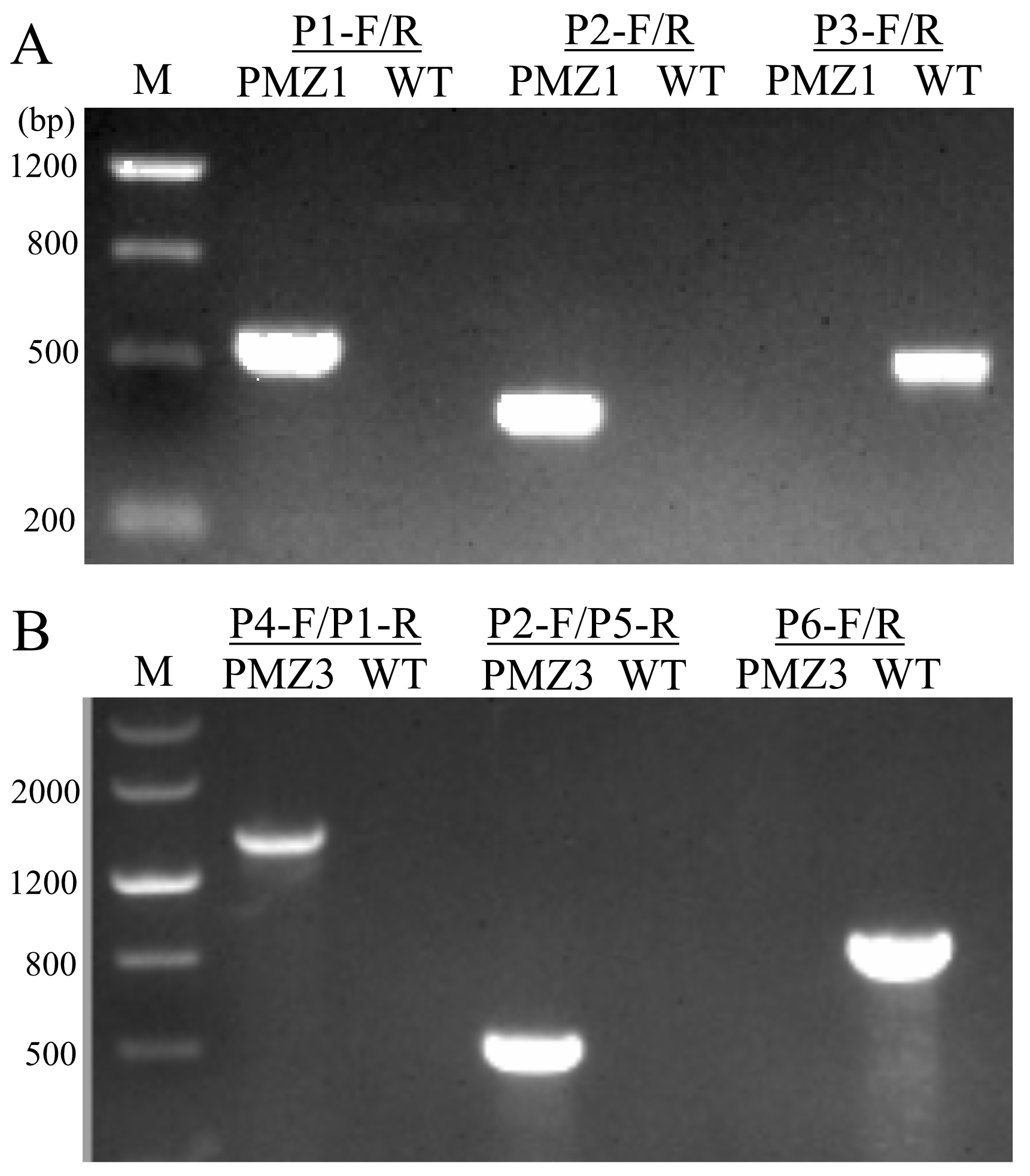

Supplement: Supplementary file 3 — Additional file 3. Characterization of the P. multocida mutant strains via PCR. The WT strain and PMZ1 (ΔpcgD) mutant were identified using primers P1-F/R, P2-F/R, and P3-F/R to confirm the pcgD mutation; similarly, the WT and PMZ3 (ΔhptE) were identified using primers P4-F/P1-R, P2-F/P5-R, and P6-F/R to confirm the hptE mutation. M refers to the DNA marker. [file 13567_2021_910_MOESM3_ESM.tiff]

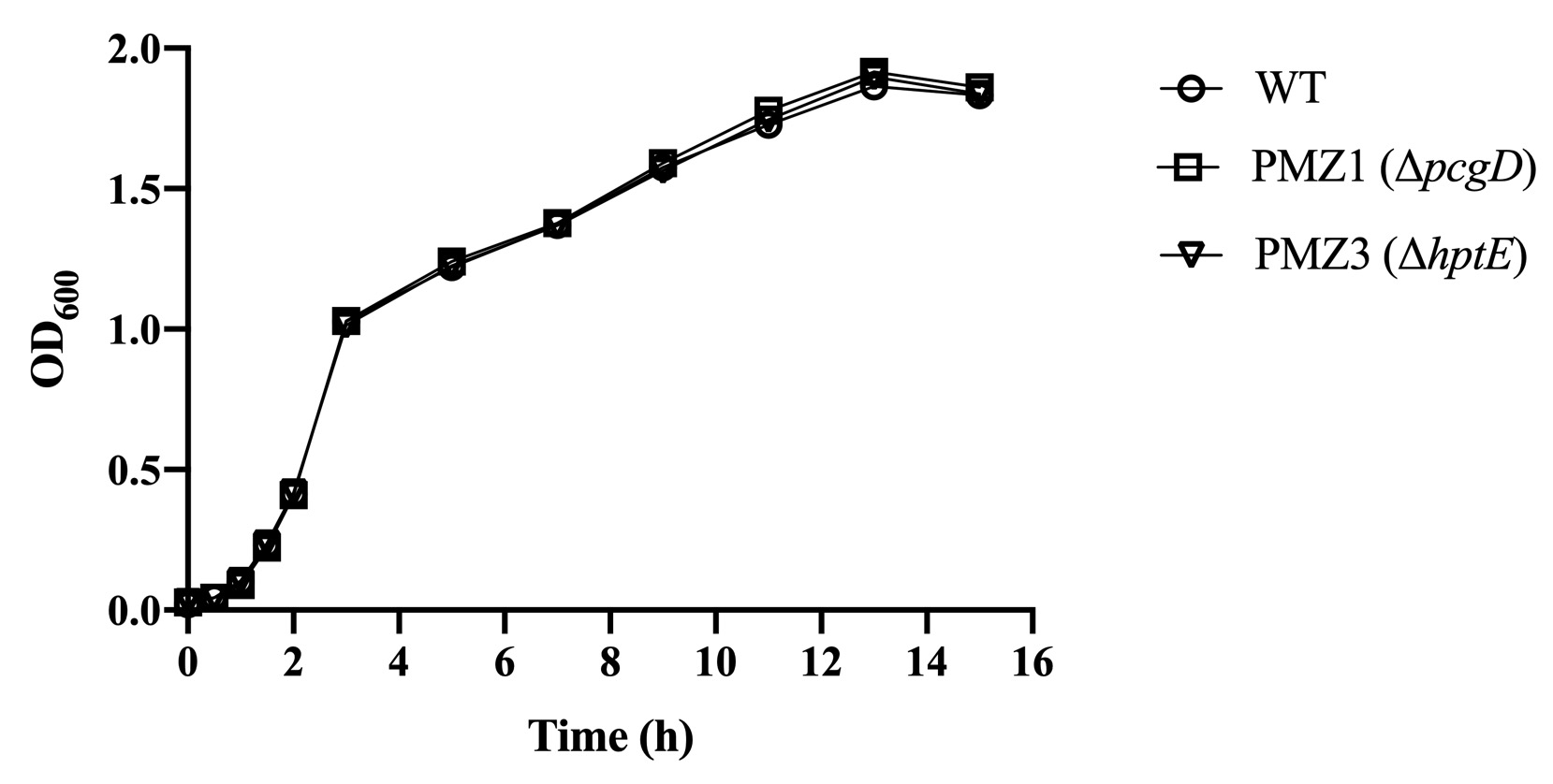

Supplement: Supplementary file 4 — Additional file 4. The growth curves. The P. multocida strains were grown in BHI medium overnight, and the cultures were diluted to an OD600 of 0.05 in fresh medium. Then the bacterial growth was determined by measuring the OD600 value every 0.5 h during the first 0 h-2 h and every 2 h from 3 to 15 h at 37 °C. [file 13567_2021_910_MOESM4_ESM.jpg]

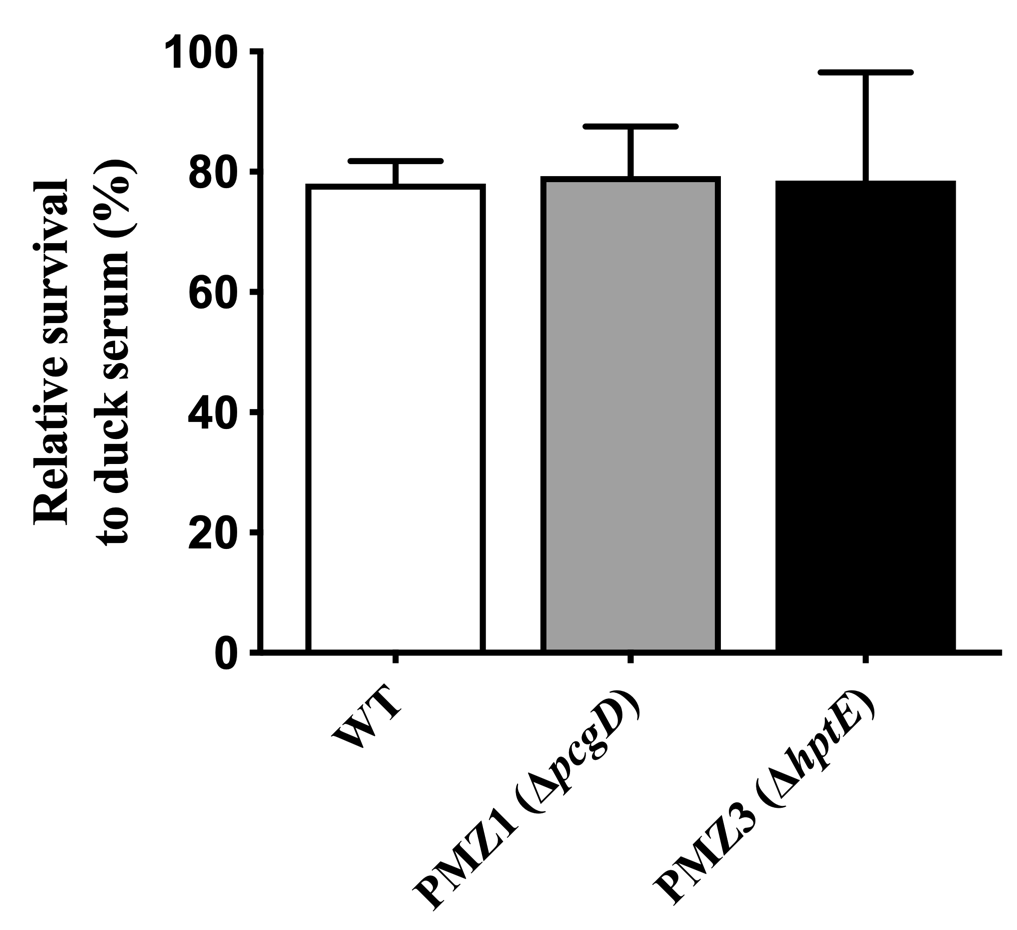

Supplement: Supplementary file 5 — Additional file 5. Serum bactericidal assay. The P. multocida strains were cultured in media to an OD600 of 0.6 and diluted to a final concentration of 104 CFU/mL in PBS. Then, the bacteria were mixed with 90% normal duck serum or heat-inactivated duck serum and incubated for 3 h at 37 °C. After incubation, serial dilutions of the samples were spread on TSA plates for counting. The survival rate of each strain was calculated as the CFU after active serum treatment divided by the CFU after the heat-inactivated serum treatment. [file 13567_2021_910_MOESM5_ESM.tiff]

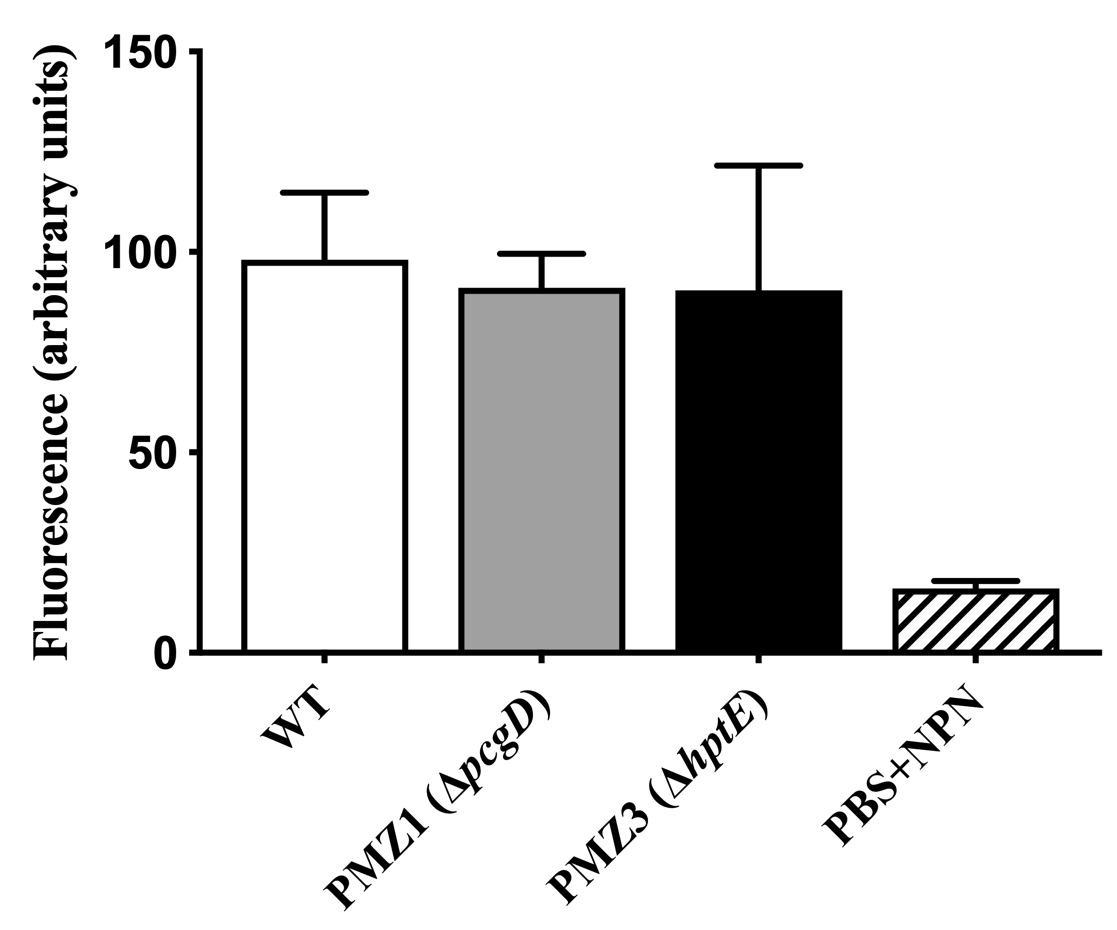

Supplement: Supplementary file 6 — Additional file 6. NPN assay. The log phase growth medium of the P. multocida strains was harvested, and the bacteria were resuspended in PBS at an OD600 of 0.5 with 80 µM NPN. PBS without bacteria was used as a control. The fluorescence of the suspension was measured immediately using a spectrofluorometer. [file 13567_2021_910_MOESM6_ESM.tiff]
