# Supplementary material for: The lipopolysaccharide outer core transferase genes pcgD and hptE contribute differently to the virulence of Pasteurella multocida in ducks
Source: Vet Res. 2021 Mar 4;52:37. doi: 10.1186/s13567-021-00910-4 (PMC7931556; doi:10.1186/s13567-021-00910-4)
Supplement: Supplementary file 7 — Additional file 7. The MICs of SDS and hydrophobic antibiotics. [file 13567_2021_910_MOESM7_ESM.docx]

**Additional file 7** **The MICs of SDS and hydrophobic antibiotics**

|  | WT | PMZ1 (Δ*pcgD*) | PMZ2 (Δ*hptE*) | ATCC25922 | |  |  |
| --- | --- | --- | --- | --- | --- | --- | --- |
|  | MIC (µg/mL) | | | |  | | |
| SDS | 128 | 128 | 256 | ＞512 | |  |  |
| novobiocin | 8 | 2 | 2 | 32 | |  |  |
| azithromycin | 2 | 2 | 2 | 4 | |  |  |
| spiramycin | 64 | 64 | 128 | 128 | |  |  |
| rifampicin | 0.5 | 0.5 | 0.5 | 16 | |  |  |
| Nalidixic acid | 64 | 32 | 32 | 4 | |  |  |
| ciprofloxacin | 2 | 4 | 4 | ＜0.25 | |  |  |
